# Calcium Chloride Activation of Acid-Washed Sewage Sludge Ash: Hydration Mechanism and Performance as a Supplementary Cementitious Material

**DOI:** 10.3390/molecules31162753

**Published:** 2026-08-07

**Authors:** Weiwei Zhu, Yi Ren, Yaxin Xiao, Shaoyu Du, Xiaoli Xie, Kao Chen

**Affiliations:** 1 School of Materials and Environment, Guangxi Key Laboratory of Advanced Structural Materials and Carbon Neutralization, Guangxi Engineering Research Center for Advanced Materials and Intelligent Manufacturing, Guangxi Minzu University, Nanning 530105, China; zhuww1230@gxmzu.edu.cn (W.Z.); zr991201@163.com (Y.R.); xyx130015@163.com (Y.X.); dusy2001@163.com (S.D.); 2 School of Civil Engineering, Guangxi Polytechnic of Construction, Nanning 530007, China; xiaoli97531@163.com; 3 College of Civil Engineering and Architecture, Guangxi University, Nanning 530004, China

**Keywords:** acid washing sludge ash, phosphorus removal, calcium chloride, volcanic ash activity activation, hydration mechanism

## Abstract

Due to the fact that municipal sludge incineration generates a large amount of toxic sewage sludge ash (SSA), cement solidification is becoming an efficient harmless treatment method. However, the large amount of phosphorus in sludge ash can delay the setting time of cement pastes and lead to a decrease in its later strength. Moreover, as an important non-renewable resource, the recycling and utilization of phosphorus is also a current research hotspot. To this end, this paper proposes to remove phosphorus from incinerated sewage sludge ash (ISSA) by the acid washing process, and, in response to the problem of reduced volcanic ash activity in acid-washed sewage sludge ash (ASSA), calcium chloride (CaCl_2_) is added as an activator to explore the optimal process and hydration mechanism for enhancing ASSA activity. This study removed phosphorus from calcined SSA (800 °C, 2 h) via acid washing (0.3 mol/L H_2_SO_4_, L/S = 10:1, 2 h) to obtain ASSA, and used CaCl_2_ as an activator to improve its pozzolanic activity. Different CaCl_2_ dosages (1–3%) were tested for paste setting time, 3/7/28 d compressive strength, and characterized by hydration heat, XRD, SEM, etc. Experimental results indicated that the synergistic addition of 2 wt.% CaCl_2_ and 20 wt.% ASSA shortened the initial and final setting times by 38% and 23% to 142 min and 289 min, respectively. Meanwhile, the compressive strength at 28 days was improved by 24%, with a final strength value of 42 MPa. Microanalysis revealed abundant Friedel’s salt formation and a denser structure (fine mesopores 60%, coarse mesopores 26%), confirming CaCl_2_ effectively activates ASSA for cement admixture use.

## 1. Introduction

Against the backdrop of the sustained economic development and the continuous advancement of urbanization, the volume of municipal sewage treatment has been increasing steadily, and the resulting sludge production has also shown a rapid growth trend [1,2]. According to the environmental statistics annual report released by the Ministry of Ecology and Environment, the sludge output of sewage treatment plants across the country reached 53.332 million tons in 2024 [3]. Sludge, a major output of sewage treatment, has a chemically complex composition. It not only has a high water content but also contains a large amount of organic matter, pathogenic bacteria and various inorganic components. Research tests show that approximately 50% of the compounds have been identified as toxic substances [4,5,6]. Therefore, it is urgent to explore efficient, safe and high-value methods for the treatment and disposal of sludge.

Currently, landfilling and incineration are the primary methods for sewage sludge disposal [7,8]. Among these, incineration has emerged as a more advantageous and promotable mainstream option due to its ability to substantially reduce sludge volume and significantly conserve land resources. However, incineration only reduces the weight of sludge by 70%, with incineration sewage sludge ash (ISSA) as the final by-product that requires further treatment [9]. Over the past few decades, such ash was typically landfilled due to its relatively small annual production volume. Nevertheless, the increasing amount of sludge being incinerated has driven the search for alternative approaches to reuse or recycle ISSA [10]. Studies have shown that ISSA, formed by high-temperature incineration of sludge, mainly consists of SiO_2_, Al_2_O_3_, P_2_O_5_, CaO, and Fe_2_O_3_ [11]. On the other hand, traditional mineral admixtures (e.g., fly ash and blast furnace slag) are facing supply shortages and rising costs, creating an urgent need to develop new alternative materials. When the contents of SiO_2_ and Al_2_O_3_ exceed 50%, ISSA exhibits comparable reactivity to fly ash after undergoing high-temperature activation, making it a potential high-quality supplementary cementitious material (SCM). In experiments conducted by Z. Chen et al. [12], the flexural strength (8.21 MPa) and compressive strength (46.74 MPa) of cement incorporating 20% ISSA were comparable to those of cement with 20% fly ash (8.08 MPa for flexural strength and 45.81 MPa for compressive strength). Y. Xia et al. [13] explored the hydration process of ternary blended cement composed of ISSA and limestone, and found that the hydration degree of C_3_S in the ternary blended cement was higher than that in the pure cement system. Additionally, ISSA applications include brick and ceramic production, phosphorus recovery, adsorbent preparation, aggregate replacement, and supplementary cementitious materials [14,15,16]. Direct utilization of ISSA in cement clinker production not only significantly reduces its disposal cost but also partially decreases the consumption of natural ore resources, offering remarkable environmental and economic benefits [17].

However, the large-scale application of ISSA in Portland cement clinker production can exert significant adverse effects on cement properties due to its high phosphorus content, such as delaying the setting time and reducing the compressive strength [6,18,19,20,21,22,23]. Notably, phosphorus is a critical element essential for biological growth and also a non-renewable strategic resource [24,25]. Some studies predict that global phosphate reserves will be depleted within the next 400 years. In China, the annual phosphorus content in wastewater is equivalent to 5.5% of the total phosphorus in national phosphate fertilizers, and approximately 90% of the phosphorus in wastewater is transferred to sludge after calcination. The phosphorus content in sludge typically accounts for 1–3% of its dry weight, and the mass concentration of orthophosphate ions in municipal wastewater in China ranges from approximately 21 to 270 mg/L [24]. If phosphorus in municipal sludge can be effectively recovered, it will not only help alleviate the pressure of phosphorus resource shortage but also enable the efficient removal of phosphorus, which holds dual significance for resource utilization and pollution control.

Internationally, the feasibility of phosphorus recovery from sewage sludge has been successfully demonstrated through various technologies [26,27]. Among these, acid leaching, a wet chemical extraction method, is one of the most commonly used phosphorus recovery approaches due to its simple working principle and no requirement for expensive equipment [28,29,30]. However, the subsequent disposal or reuse of acid-leached sewage sludge ash (ASSA), the solid residue remaining after phosphorus recovery, has received relatively limited research attention. Kappel et al. [31] replaced 20% of cement with electrodialytically treated SSA. Their findings showed that while the treated sludge did not cause a decrease in cement compressive strength, it significantly reduced mortar workability, failing to balance strength and workability simultaneously. Ottosen et al. [29] analyzed the feasibility of utilizing ASSA in building materials. Building on this research, the present study first investigated the potential of using ASSA as a 10% sand replacement material. The results show that due to the characteristics of ASSA, the demand for mixed water increases, and thus the effect is poor. Subsequently, this study further evaluated the feasibility of directly replacing cement with ASSA. The results demonstrated that replacing 20% of cement with ASSA yielded a compressive strength of 52 MPa, which was lower than that of the control group (70 MPa). In conclusion, these studies provide strong support for the feasibility of ASSA as a cementitious material for cement mortar. Acid leaching can effectively dissolve phosphorus and also remove or stabilize metal ions through complexation, thereby reducing environmental risks. Nevertheless, the acid leaching process also consumes active components in ISSA (e.g., free CaO, C_3_S) and lowers its pH value; this damages the early hydration and strength development of cement [29,32]. To mitigate the adverse effects caused by acid leaching, exploring strategies to enhance the pozzolanic activity of ASSA is a topic worthy of further investigation.

To enhance the volcanic ash activity of SSA, the introduction of a chemical activator such as alkali excitation, sulfate excitation and compound excitation has become one of the most effective methods for enhancing the activity of sludge ash [33,34,35,36]. However, acid-washed treatment will completely change the mineral crystal form, elemental composition and phosphorus combination form of ISSA, resulting in significant differences in the physical and chemical properties and structural characteristics of ASSA from conventional sludge ash. Traditional mature activation processes cannot meet the modification requirements of ASSA and have difficulty explaining the activation rules of sludge ash under pickling. Among all the inorganic activators, NaCl was superior to MgCl_2_ because NaCl contains more chloride ions, resulting in more Friedel’s salts. Moreover, its solution has a higher alkalinity, which can effectively lead to a gradual increase in the pozzolanic reaction for SSA [35,36]. However, due to the low calcium content in ISSA, the overall activation effect was not as good as that of CaCl_2_ and CaSO_4_. Q. X. Zhao et al. [37] compared the activation effects of CaCl_2_ and CaSO_4_ on the SSA and found that the activation effect of CaCl_2_ was better than that of CaSO_4_. This was mainly attributed to the fact that the Cl^−^ dissolved in the solution would react with Ca^2+^ to form Ca(ClO)_2_ precipitation, accelerating the release of calcium ions and thus accelerating the cement hydration process. Moreover, the increase in solution alkalinity also activated the pozzolanic reaction of SSA. These studies were based on the reports of ISSA, but there were few studies on the activation of ASSA. In conclusion, CaCl_2_ is inexpensive and can make up for the calcium ion loss caused by acid washing, which may be of great help in the activity of ASSA.

Previous studies on chloride activation have predominantly focused on pure slag [37] or fly ash systems [38], leaving three critical unknowns in the context of ASSA: (i) whether CaCl_2_ can effectively offset the strength loss caused by acid washing without compromising workability; (ii) what the optimal CaCl_2_ dosage is for balancing early-age hydration and long-term microstructure development in ASSA-blended cements; and (iii) how the coupled effect of CaCl_2_ and residual phosphorus species influences the hydration kinetics and phase assemblage (particularly Friedel’s salt formation) in a way that differs from conventional chloride-activated systems. To address these gaps, this study systematically investigates the hydration mechanism and performance of ASSA–cement pastes modified with varying CaCl_2_ dosages (1–3 wt.%). Firstly, the effects of CaCl_2_ on the macroscopic properties of the paste are clarified through setting time tests and compressive strength tests. Subsequently, the evolution law of hydration products and the development characteristics of micro-morphology within the cement are explored in depth via hydration heat analysis, X-ray diffraction (XRD), Fourier transform infrared spectroscopy (FTIR), Mercury intrusion porosimetry (MIP), thermogravimetry–differential scanning calorimetry (TG-DSC), and scanning electron microscopy (SEM) tests. Finally, combined with the variation laws of macroscopic properties, hydration products, and micro-morphology, the feasibility of ASSA–cement as a construction material is comprehensively evaluated. This study aims to provide a new technical pathway for the resource utilization of industrial solid wastes. The findings are expected to contribute to both the sustainable management of incineration residues and the development of high-performance blended cements with reduced environmental footprint.

## 2. Results

### 2.1. Setting Time and Volume Stability

**Figure 1:**
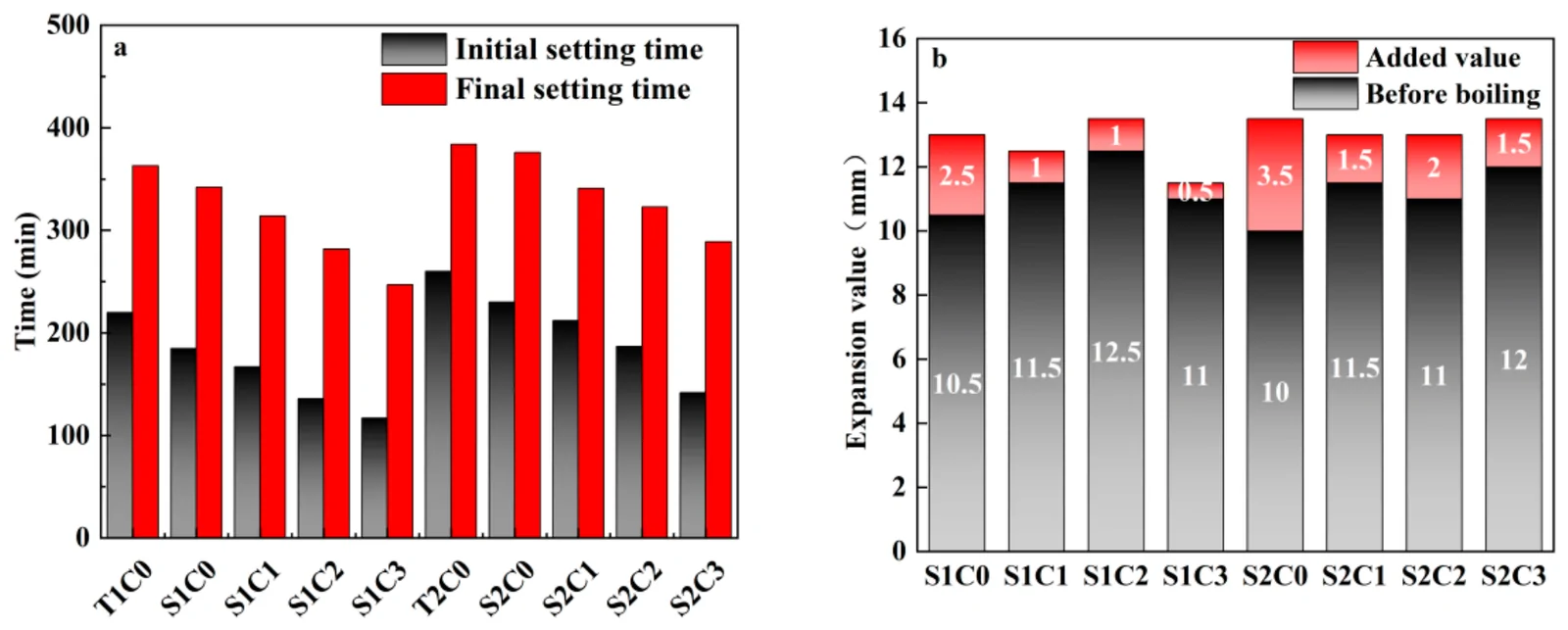
(**a**) Cement paste setting time. (**b**) Volume stability.

Figure 1a,b show the effects of different dosages of ASSA and CaCl_2_ on the initial setting time, final setting time, and volume stability of ASSA–cement pastes, respectively. Compared with the effect of acid washing on SSA, the results show that the setting time after acid washing is significantly shortened. Taking S1C0 and S2C0 without CaCl_2_ addition as the control groups, with the increase in ASSA dosage, both the initial and final setting times of the cement pastes exhibited an increasing trend. The initial setting time was prolonged from 185 min to 230 min, and the final setting time was extended from 342 min to 376 min. This indicates that the increase in ASSA dosage is detrimental to cement hardening, which may be attributed to the decrease in the relative content of cement clinker and thus the reduction in active substances caused by the increase in ASSA dosage [13]. Based on the blank group S2C0 without CaCl_2_ addition (initial setting time: 230 min, final setting time: 376 min), the increase in CaCl_2_ dosage gradually shortened the initial and final setting times, with the effect becoming more significant at higher concentrations. Specifically, for group S2C3, the initial setting time was 142 min and the final setting time was 289 min, which decreased by approximately 40% and 23%, respectively, compared with the blank group. This suggests that the addition of CaCl_2_ can promote the hydration of cement pastes, which is consistent with the findings reported in previous studies [39,40]. As shown in Figure 1b, the volume stability of all cement pastes was not more than 5.0 mm. The results of setting time and soundness complied with the requirements of the relevant requirement, which specifies that the initial setting time of all cements shall not be earlier less 45 min, the final setting time shall not be more than 390 min, and the volume stability shall not exceed 5.0 mm.

### 2.2. Compressive Strength

**Figure 2:**
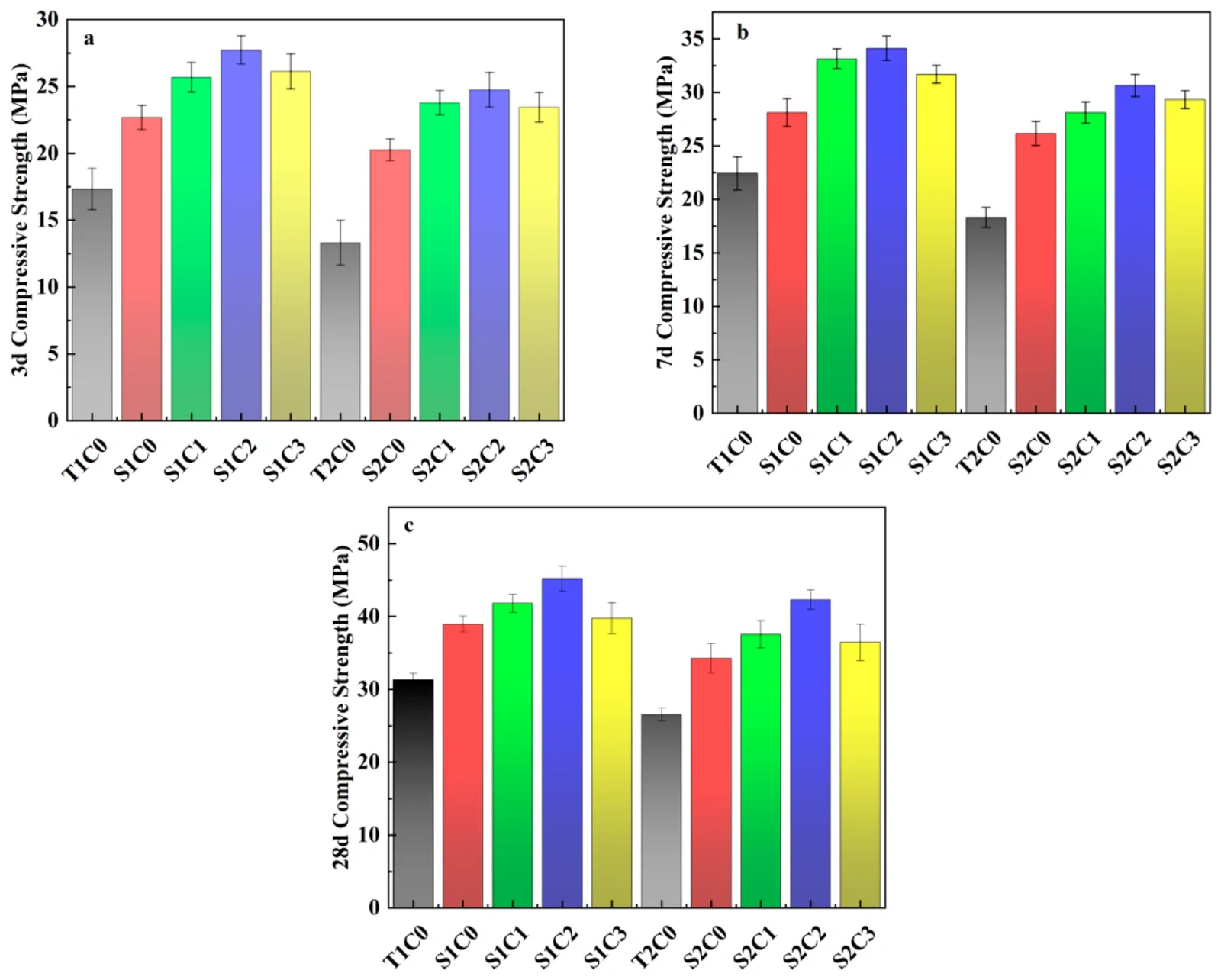
Effects of different dosages of ASSA and CaCl_2_ on the compressive strength of cement mortar, (**a**) 3-day curing age; (**b**) 7-day curing age; (**c**) 28-day curing age.

The effects of different dosages of ASSA and CaCl_2_ on the compressive strength development of cement mortar specimens with curing age are illustrated in Figure 2. Compared with SSA, the ASSA exerts a more significant enhancing effect on the compressive strength of cement. For cement incorporating 20% ASSA, the compressive strength at the 28-day curing age increased from 26 MPa to 34 MPa, indicating that acid leaching treatment can effectively enhance the reactivity of the SSA. Comparing mortars with different ASSA dosages, it can be observed that with the increase in ASSA dosage, the compressive strength exhibits a significant decreasing trend. The 28-day compressive strength decreased from 39 MPa to 34 MPa, with a decrease of approximately 20%, which is consistent with the trends reported in the existing literature [29]. After the incorporation of CaCl_2_ into the cement, the overall compressive strength was improved. When the dosage of CaCl_2_ is ≤2%, the compressive strength increases with the increase in its dosage. However, when the dosage exceeds 2%, the strength shows a downward trend. For example, the 28-day compressive strength of group S2C3 was 36 MPa, which was lower than that of group S2C2 (42 MPa). This phenomenon may be related to the fact that CaCl_2_ promotes the formation of a large amount of ettringite (AFt), resulting in volume expansion [41]. Since CaCl_2_ exhibits a significant reinforcing effect at an ASSA dosage of 20%, subsequent studies will focus on this dosage level to further investigate the mechanism of CaCl_2_ on the ASSA–cement through the analysis of the hydration process and microstructure.

### 2.3. Isothermal Calorimetry

**Figure 3:**
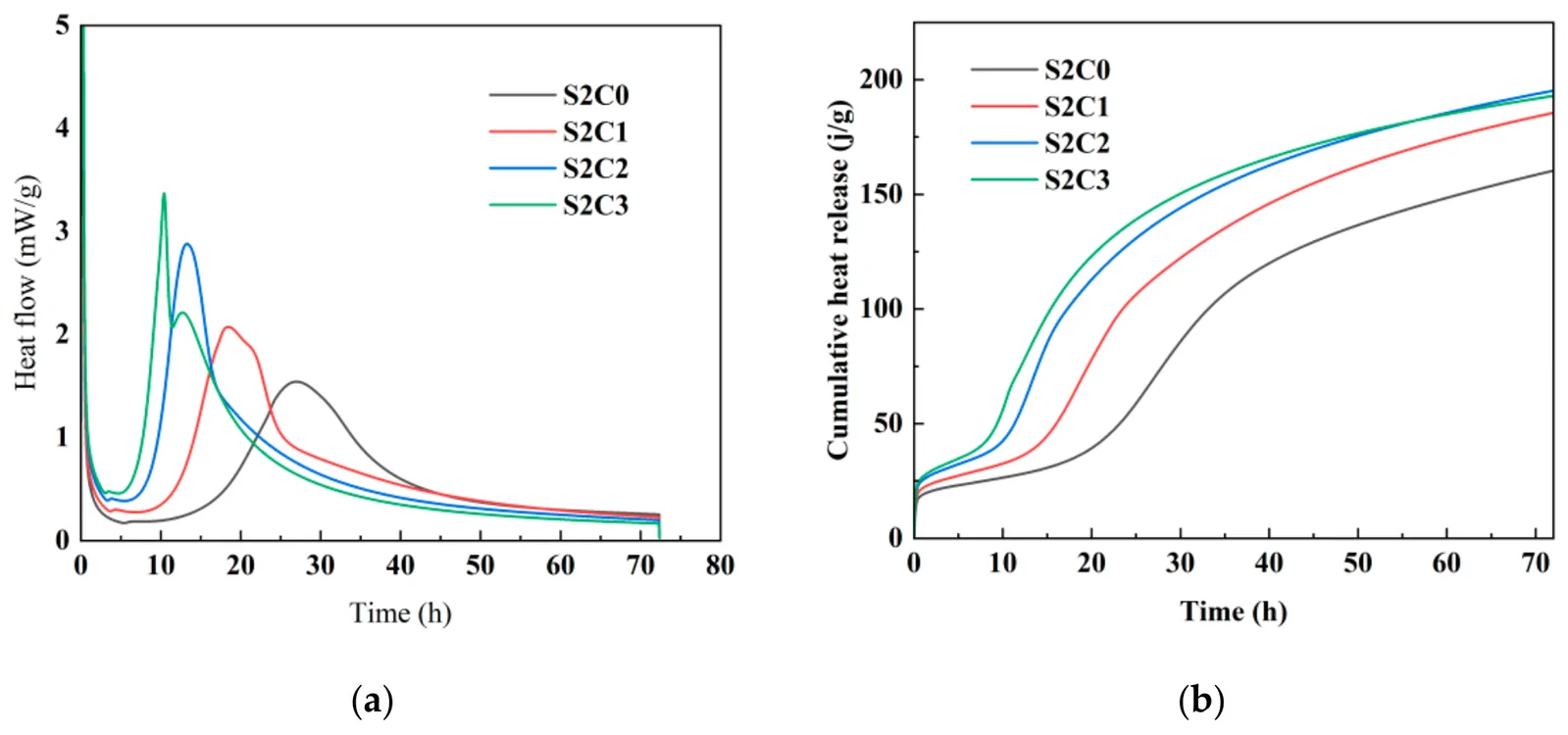
Hydration heat of 20% ASSA–cement excited by different dosings of CaCl_2_: (**a**) heat release rate; (**b**) cumulative heat release.

The hydration process of cement pastes was evaluated via isothermal calorimetry, with the results presented in Figure 3. Figure 3a illustrates the effects of different CaCl_2_ dosages on the hydration process of cement pastes containing 20% ASSA. The cement hydration reaction can be divided into five stages: the pre-induction period, induction period, acceleration period, deceleration period, and stabilization period. In the control group S2C0 without CaCl_2_ addition, the hydration induction period was the longest and the intensity of the main hydration peak was the lowest, whereas with the incorporation of CaCl_2_, the hydration induction period of cement pastes was significantly shortened, and the intensity of the main hydration peak gradually increased with the increase in CaCl_2_ dosage. Since the main hydration peak mainly corresponds to the hydration reaction of C_3_S, the above results indicate that CaCl_2_ can promote the hydration of C_3_S in the 20% ASSA–cement and shorten the induction period, which is also consistent with the improvement in compressive strength. In addition, a second hydration peak appeared in group S2C3 (around 15 h) [9,42], which may be attributed to the reaction of CaCl_2_ with cement to form Kuzel’s salt and Friedel’s salt. Figure 3b shows the variation in cumulative heat release of the 20% ASSA–cement with different CaCl_2_ dosages. It can be observed that group S2C3 released the most heat in a relatively short time, and the cumulative heat release continued to increase, indicating that its hydration reaction was both rapid and persistent, with the highest total heat release among the four groups; in contrast, the cumulative heat release of group S2C0 increased most slowly, further confirming that its hydration process was relatively slow. It is worth noting that in the later stage of the test, the heat release of group S2C2 gradually approached that of group S2C3, which may be the reason why the compressive strength of S2C2 exceeded that of S2C3.

### 2.4. XRD Analysis

**Figure 4:**
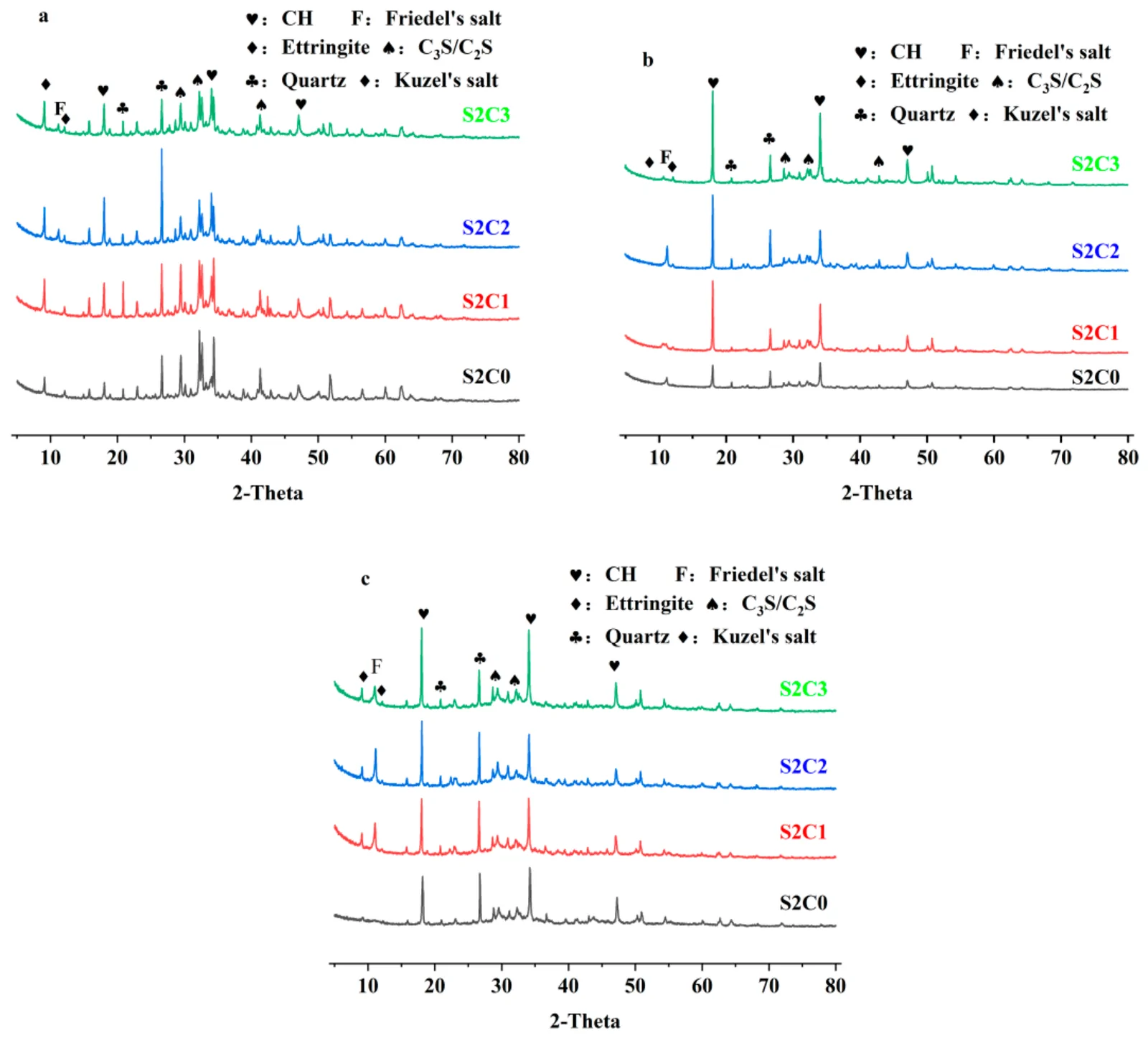
XRD of 20% ASSA–cement pastes at different ages with different dosages of CaCl2: (**a**) 1 day, (**b**) 7 days, (**c**) 28 days.

XRD analysis was conducted on the 20% ASSA–cement at different curing ages (1 day, 7 days, 28 days), with the results depicted in Figure 4a–c. The degree of cement hydration can be reflected by the intensity of characteristic diffraction peaks. The analysis reveals that Ca(OH)2, AFt, and a small amount of C-S-H gel are the primary hydration products in the early stage of hydration. Meanwhile, the incorporation of CaCl_2_ triggers reactions with cement to form Kuzel’s salt and Friedel’s salt. This phenomenon can be attributed to the replacement of sulfate ions in the AFm phase by chloride ions as CaCl_2_ dosage increases: Kuzel’s salt is formed at low chloride ion concentrations, whereas Friedel’s salt is generated at higher concentrations. In the control group (S2C0) without CaCl_2_ addition, the characteristic peak intensities of C_3_S and C_2_S are the highest. Dissociated sulfate ions react with Ca^2+^ and Al^3+^ to form AFt. With the incorporation of CaCl_2_, the characteristic peak intensities of C_3_S and C_2_S gradually decrease, indicating that CaCl_2_ promotes the consumption of unhydrated clinker minerals. The control group exhibits the highest diffraction peak intensity of CH; however, at early curing ages (e.g., 1 day), its AFt characteristic peak intensity is the lowest. In contrast, groups with CaCl_2_ addition show stronger AFt peaks, demonstrating that Cl^−^ can accelerate the reaction between C_3_A and gypsum, thereby promoting AFt formation. In summary, the addition of CaCl_2_ significantly accelerates the hydration process of the 20% ASSA–cement. Cl^−^ plays a dual role: on the one hand, it expedites the reaction between C_3_A and gypsum to form AFt; on the other hand, it facilitates the hydration of C_3_S and C_2_S, leading to the generation of more CH and C-S-H gel, accompanied by the formation of Friedel’s salt. These observations are consistent with the findings when CaCl_2_ and sodium chloride were used to activate the sludge ash [36,38]. The combined effects of these changes not only enhance the compressive strength but also shorten the setting time of the cement.

### 2.5. TG-DTG

On the DTG/TG curves, the following thermal decomposition behaviors were observed: the mass loss in the range of 70–100 °C was attributed to the decomposition of AFt, and the mass loss between 150 and 400 °C resulted from the decomposition of Friedel’s salt. The mass loss in the range of 400–520 °C was associated with the decomposition of CH, while the mass loss between 650 and 780 °C was related to the decomposition of calcite.

**Figure 5:**
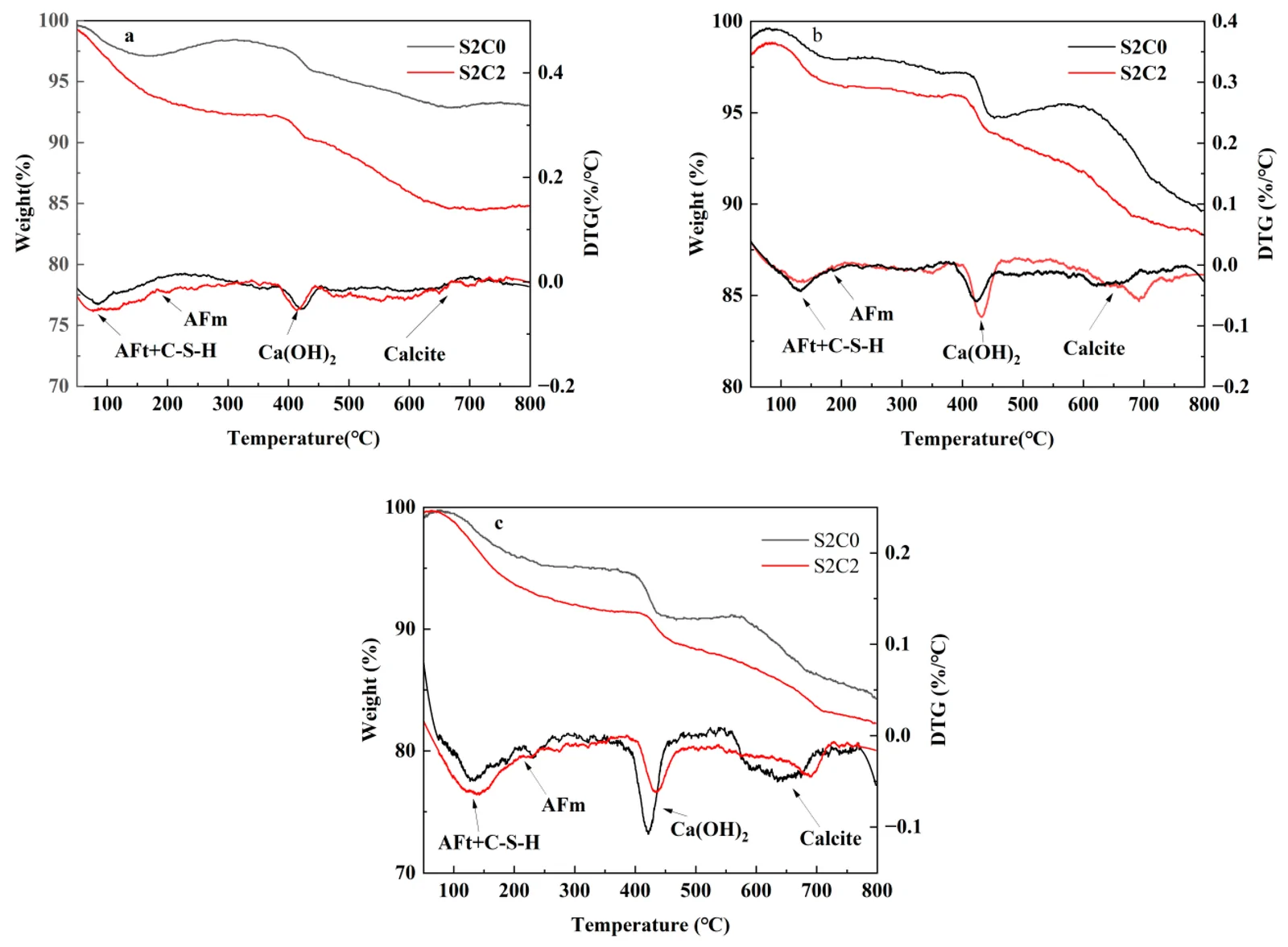
TG-DTG of 20% ASSA–cement pastes at different ages with different dosages of CaCl_2_: (**a**) 1 day; (**b**) 7 days; (**c**) 28 days.

Figure 5 presents the TG-DTG test results of the samples at curing ages of 1 day, 7 days, and 28 days. According to the thermogravimetric curves, the mass loss could be divided into four stages. With the increase in CaCl_2_ dosage, the total mass loss of the samples increased continuously. By comparing samples with different CaCl_2_ dosages, the difference in mass loss rate before 400 °C increased significantly, indicating that the incorporation of CaCl_2_ promoted the formation of hydration products and accelerated the hydration process. This was consistent with the phenomenon observed in the XRD tests: the addition of CaCl_2_ increased the content of hydration products and improved the degree of hydration. Meanwhile, a comparison of the test results at different curing ages revealed that at later ages, the CH content of the control group was significantly lower than that of the samples with CaCl_2_ addition. This phenomenon was also consistent with the XRD results, both indicating that the incorporation of CaCl_2_ enhanced the hydration reaction of the paste.

### 2.6. FTIR Analysis

**Figure 6:**
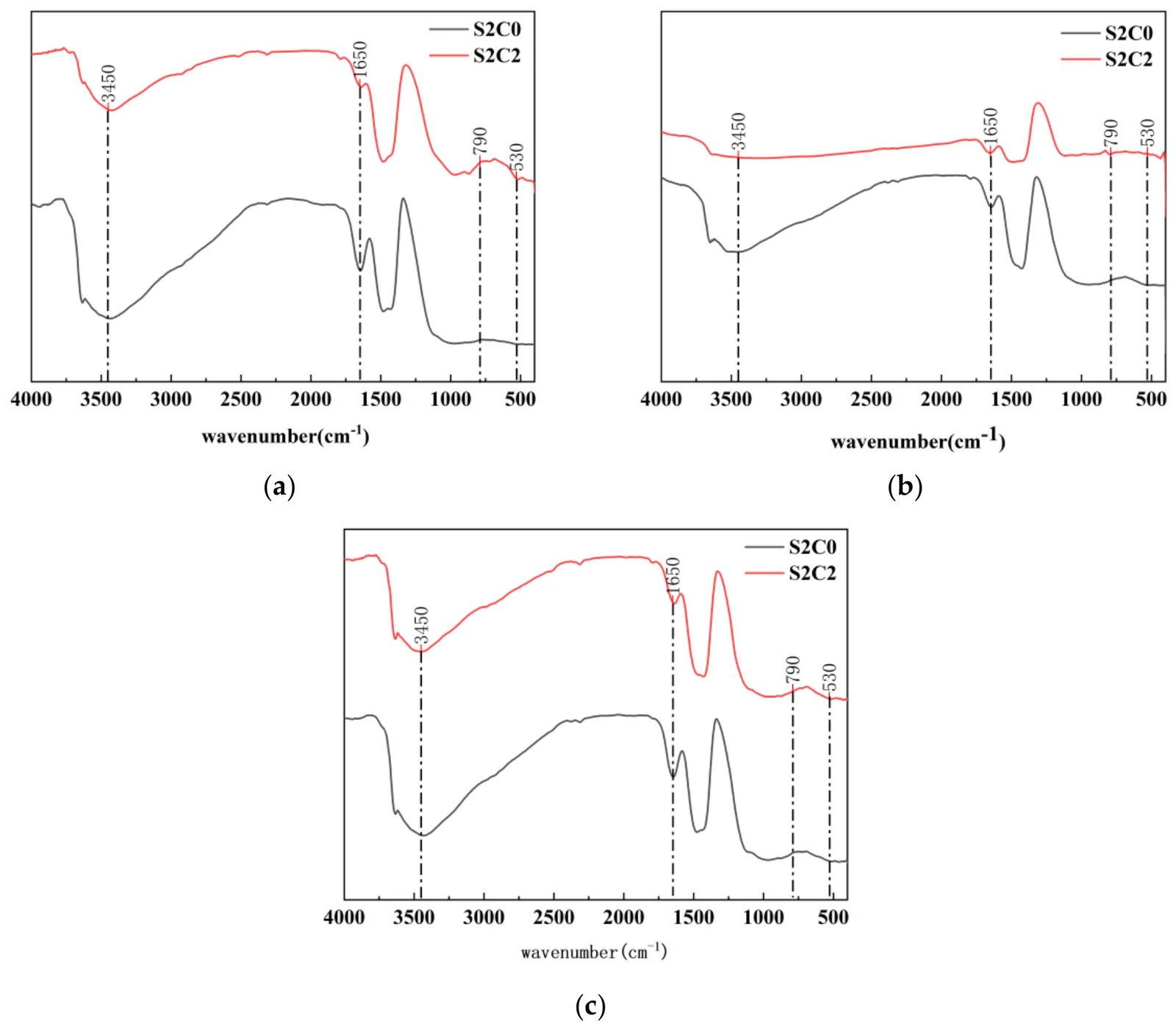
FTIR of 20% ASSA–cement pastes at different ages with different dosages of CaCl2: (**a**) 1 day, (**b**) 7 days, (**c**) 28 days.

Figure 6 presents the effects of different CaCl_2_ dosages on the FTIR spectra of the 20% ASSA–cement system at various curing ages (1 day, 7 days, and 28 days), where Figure 6a–c correspond to the curing ages of 1 day, 7 days, and 28 days, respectively. The bands at 530 cm^−1^ and 790 cm^−1^ are attributed to the bending and stretching vibrations of the Al–O–H bond in Friedel’s salt, respectively; when the CaCl_2_ dosage is ≥2%, the intensities of these two characteristic peaks increase. The bands near 3450 cm^−1^ and 1650 cm^−1^ arise from the stretching and bending vibrations of the O–H bond in the structural water of hydration products. The intensities of all these peaks gradually increase with the incorporation of CaCl_2_. These phenomena indicate that the proportion of the amorphous phase in the material increases correspondingly when the CaCl_2_ dosage is 2%. The improvement in the content of amorphous products results in a denser pore structure, which in turn provides favorable conditions for the development of compressive strength.

### 2.7. MIP

**Figure 7:**
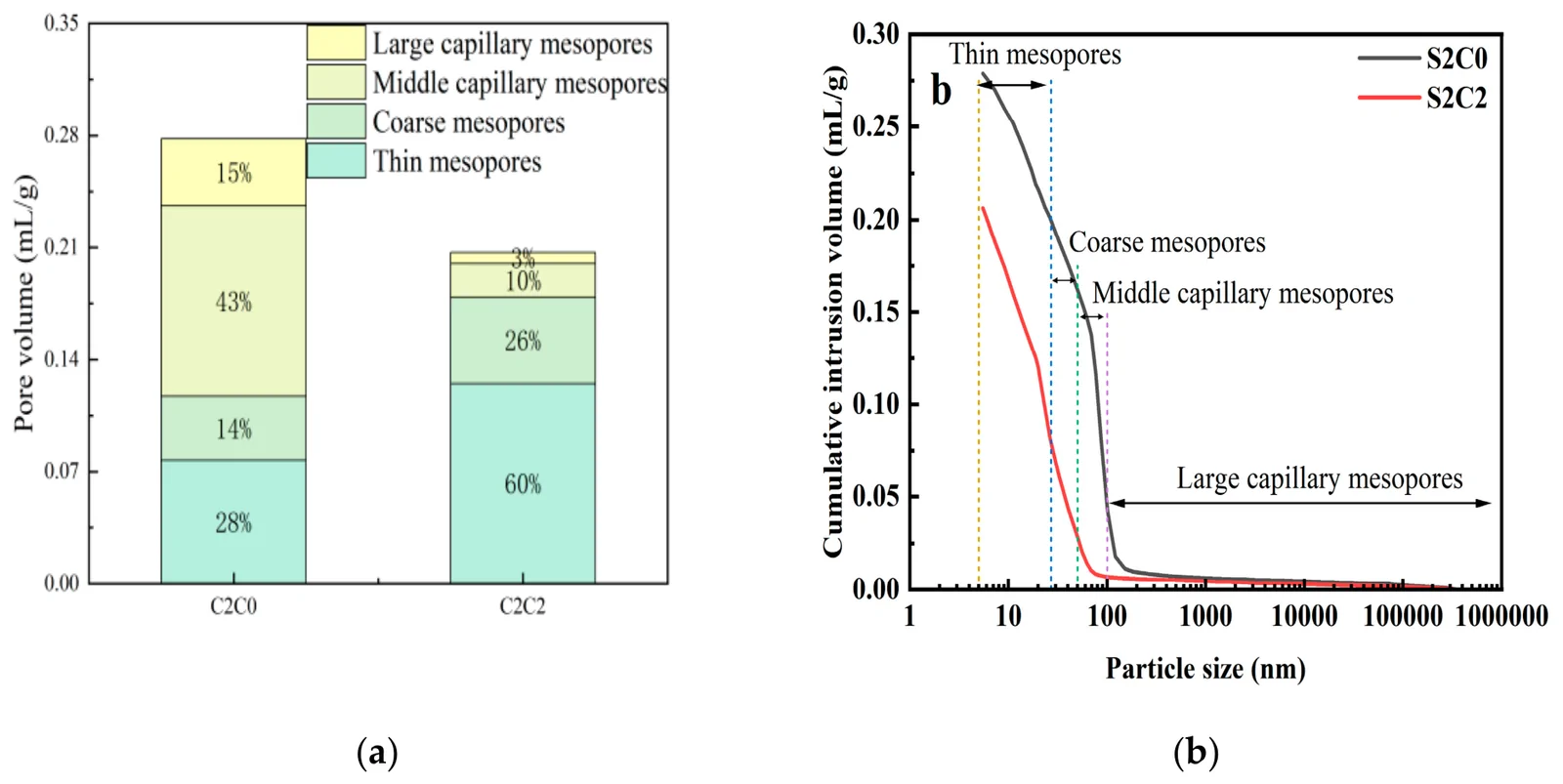
Pore structure of S2C0 and S2C2 after 28 days of curing: (**a**) cumulative pore volume curve and (**b**) pore volume and proportion of various pore types in S2C0 and S2C2.

In this study, the pore structure of the cement-based composites was classified into four categories: thin mesopores (5–27 nm), coarse mesopores (27–50 nm), medium capillary mesopores (50–100 nm), and large capillary mesopores (>100 nm). The research focused on investigating the effects of CaCl_2_ on the porosity and pore size distribution of the 20% ASSA–cement. Previous studies have shown that pores in the range of 100–1000 nm are detrimental to the mechanical strength of cement-based materials in MIP measurements [43]. As shown in Figure 7a, the total porosity of the material decreased significantly after the incorporation of CaCl_2_. This can be attributed to the two main effects of CaCl_2_: on the one hand, it accelerates the cement hydration reaction. On the other hand, chloride ions react with hydration products such as C_3_A to form Friedel’s salt, which fills the pores and improves compactness, ultimately leading to a decrease in total porosity. While Figure 7b illustrates that the pore structure of the control group is dominated by medium capillary pores, with low proportions of fine mesopores and coarse mesopores, the situation is significantly different after the incorporation of CaCl_2_. The proportion of fine mesopores increased from 28% to 60%, and that of coarse mesopores increased from 14% to 26%. The volume proportions of fine mesopores and coarse mesopores increased significantly and were much higher than those of the control group; thus the compressive strength of the samples with CaCl_2_ addition was much higher than that of the control group.

## 3. Discussion

**Figure 8:**
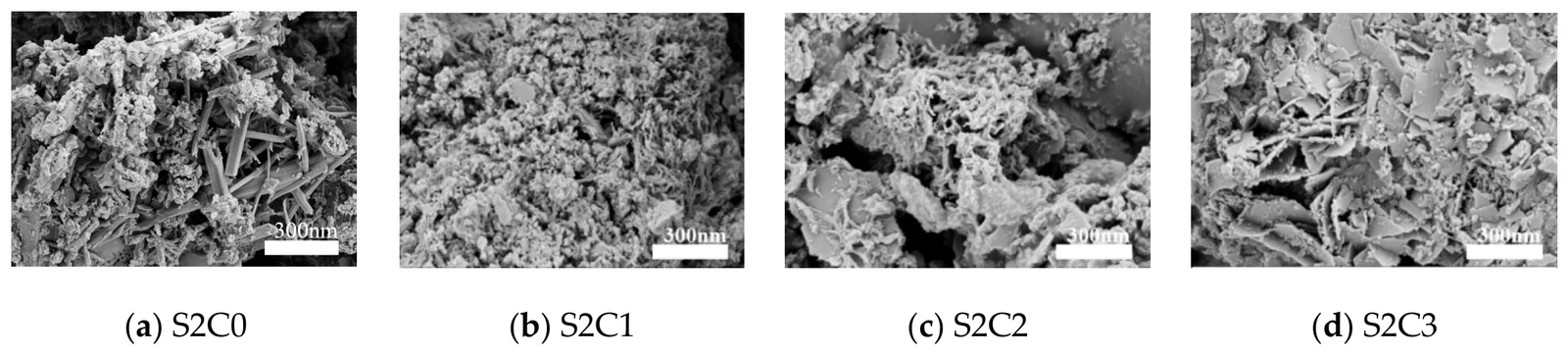
The microstructure of 20% ASSA–cement at different dosings of CaCl_2_ at 1 day.

**Figure 9:**
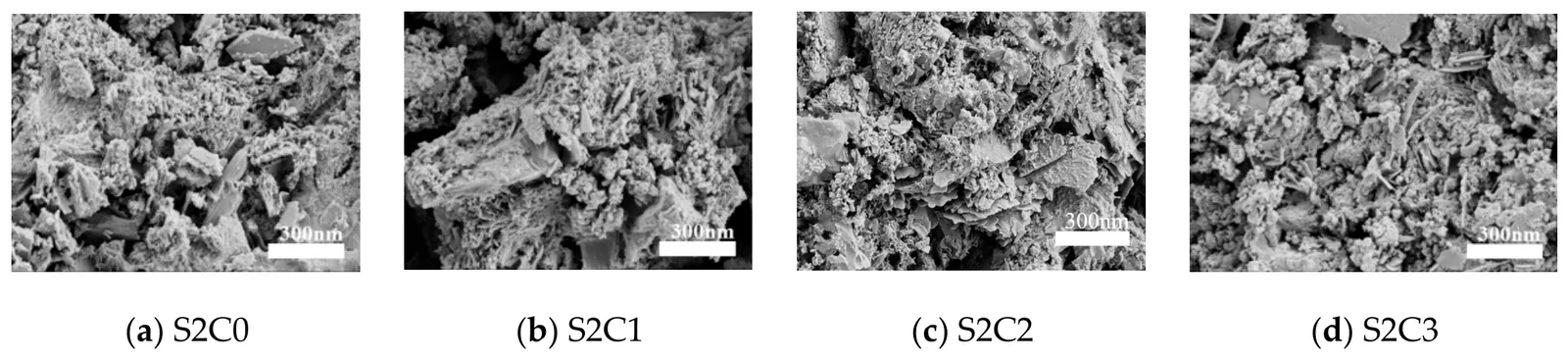
The microstructure of 20% ASSA–cement with different dosings of CaCl_2_ at 7 days.

**Figure 10:**
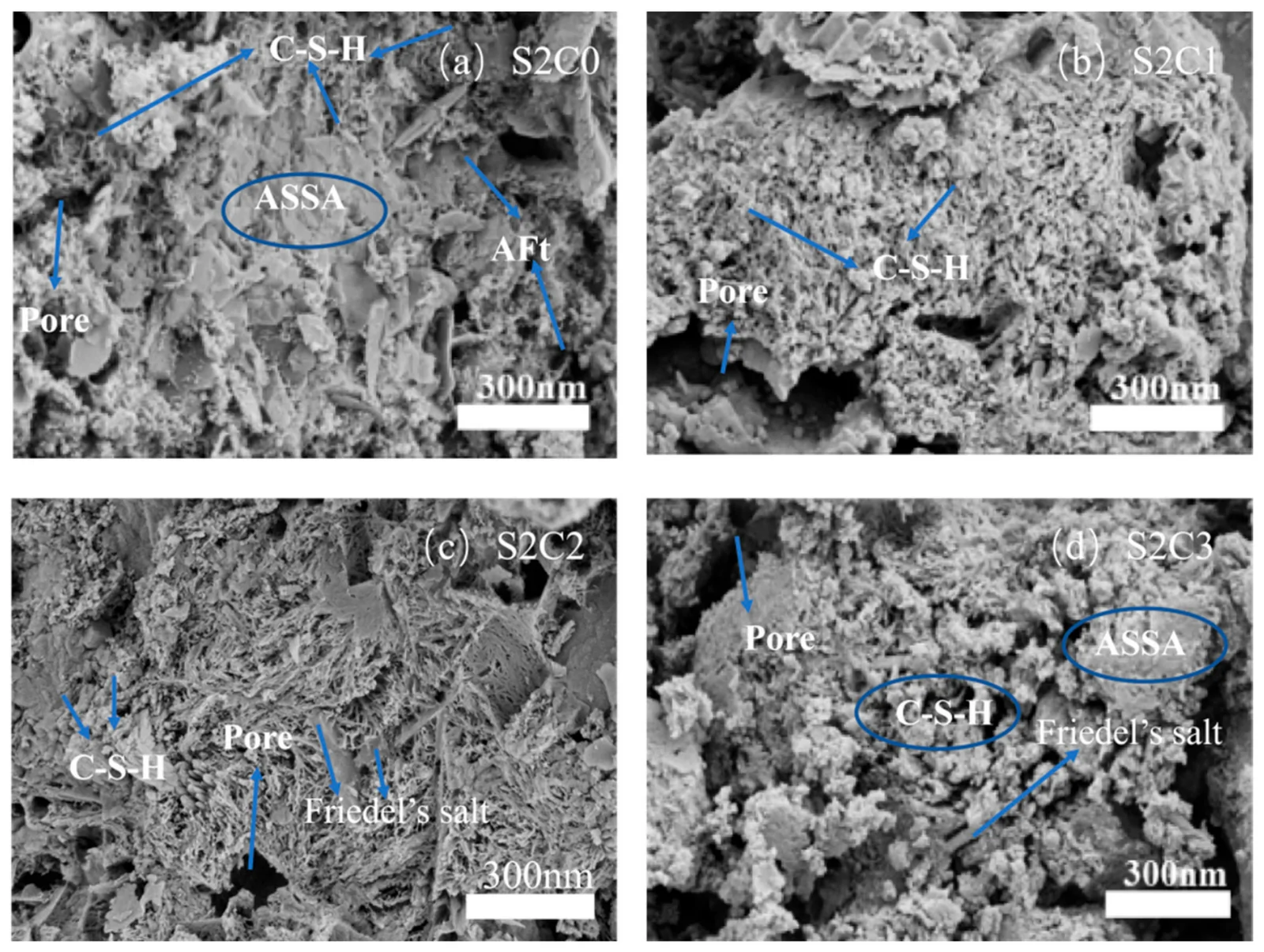
The microscopic morphology of 20% ASSA–cement with different dosings of CaCl_2_ at 28 days.

SEM was employed to analyze the microstructural morphology of the 20% ASSA–cement with different CaCl_2_ dosages (Figure 8, Figure 9 and Figure 10 correspond to curing ages of 1 day, 7 days, and 28 days, respectively). At the 1-day curing age, the S2C0 group without CaCl_2_ addition exhibited a loose structure with numerous voids and distinct unhydrated particles. In contrast, the S2C1, S2C2, and S2C3 groups presented a denser structure with reduced pores and unhydrated products, accompanied by the formation of a large number of acicular and prismatic AFt crystals and flocculent amorphous C-S-H gel. At 7 days, only partial surfaces of particles in the S2C0 group were covered by hydration products. The S2C1 and S2C2 groups displayed a uniform structure with filled pores, and hexagonal plate-like Friedel’s salt was observed simultaneously. However, the S2C3 group exhibited a loose surface with pores. At 28 days, the porosity of the S2C0 group decreased significantly, with C-S-H gel fully filling the pores. The S2C1 and S2C2 groups achieved a denser structure, while internal microcracks appeared in the S2C3 group, indicating that excessive CaCl_2_ is detrimental to strength development. This is consistent with the phenomenon observed in the compressive strength tests, where the strength decreased when the CaCl_2_ dosage exceeded 2%. The cause of this phenomenon can be attributed to the fact that the calcium chloride content reaches 3%, and a large amount of chloride ions penetrate and react with the C3A to form a large amount of Friedel salts, which continuously generate and aggregate in the confined pore space. When the pressure generated by the crystal growth exceeds the material’s bearing capacity, microcracks will form around the pores [38]. This result is consistent with the XRD results. XRD also shows that, compared with a lower calcium chloride content, when 3% of calcium chloride is added, the amount of Friedel salts is greater and the amount of AFt is smaller.

**Figure 11:**
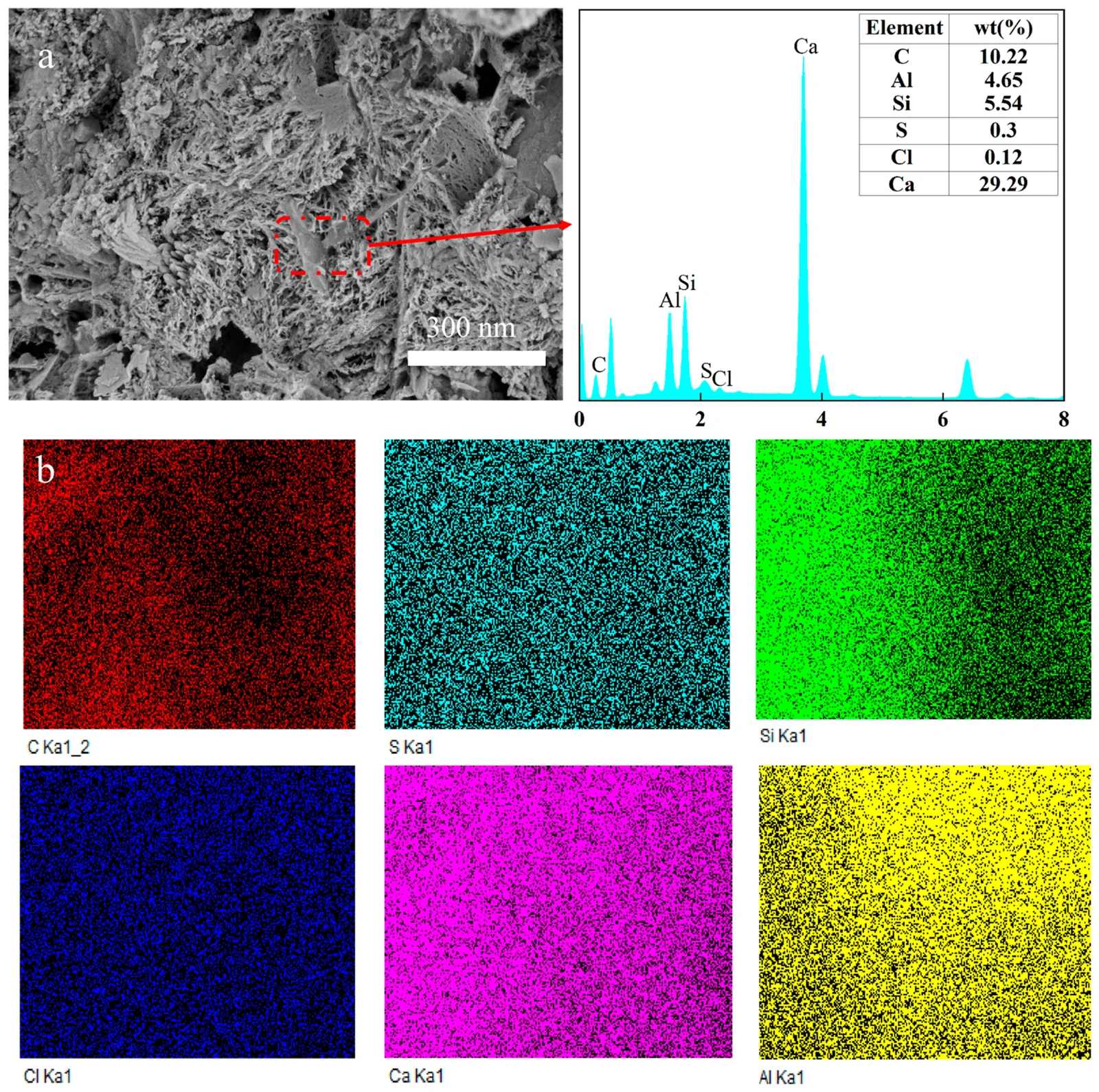
(**a**) SEM of S2C2; (**b**) EDS (28 d) of S2C2.

EDS analysis was performed on the S2C2 sample, with the results presented in Figure 11. Friedel’s salt exhibits a hexagonal plate-like structure with a size of approximately 2–3 μm. The EDS results indicated that this substance mainly contains Ca, Al, and Cl elements, which is consistent with the main chemical composition of Friedel’s salt. We further analyzed the Ca/Si ratio and the Cl/Al ratio and found that the Ca/Si ratio was 3.72 and the Cl/Al ratio was 0.48. The measured Ca/Si ratios appear higher than expected for pure C-S-H, and the Cl/Al ratios deviate from the theoretical stoichiometry of Friedel’s salt (Ca:Al:Cl = 2:1:1). The phenomenon of elevated Ca/Si ratios (3.72 in our EDS point analyses) is attributed to the composite nature of the analyzed micro-regions. As shown in Figure 11, our EDS point analyses were performed on areas containing not only C-S-H gel but also unhydrated cement clinker grains (C_3_S and C_2_S) and partially reacted ASSA particles. These phases intrinsically possess high Ca contents and low Si contents, which inevitably elevate the bulk Ca/Si ratio of the analyzed volume—a well-documented artifact in EDS analysis of cementitious materials. On the other hand, regarding the lower-than-expected Cl/Al ratios (0.48 vs. the theoretical 1.0 for pure Friedel’s salt), we theorize that this discrepancy arises because the EDS probe interacts with a multi-phase mixture rather than pure Friedel’s salt crystals. The Friedel’s salt crystals observed are typically <1 μm in size and are intimately intermixed with C-S-H gel, AFt, and unhydrated particles (see Figure 11). Consequently, the Al and Cl signals from Friedel’s salt are diluted by the surrounding Ca- and Si-rich phases.

M. C. G. Juenger et al. [44] used the CaCl_2_ to activate the C_3_S, and found that the calcium chloride could accelerate the hydration of C_3_S and produced more hydration products. This finding is consistent with our findings. Further comparison was made using CaCl_2_ as the activator to stimulate the SSA. We found that Q. X. Zhao et al. [37] replaced 50% of the cement with 50% sludge ash and added 1%, 2%, and 3% of calcium chloride. The 7-day compressive strength increased by 41.4%, 49.8%, and 39.8%, respectively. This result is consistent with the best working performance achieved by adding 2% CaCl_2_ in this paper.

The Friedel’s salt formation observed in SEM and XRD indicates chemical binding of chlorides, which can partially reduce the concentration of free chloride ions in the pore solution. In addition, the refined pore structure (60% fine mesopores) induced by 2% CaCl_2_ addition may actually slow down the ingress of external harmful ions (e.g., Cl^−^, SO_4_^2−^, CO_2_) due to reduced capillary connectivity. This intrinsic densification could provide a certain level of compensatory protection against durability degradation [45]. However, we fully recognize that chloride binding is reversible—under carbonation or sulfate attack, bound chlorides may be released back into the pore solution, potentially increasing the corrosion risk over time.

The total chloride content in the 2 wt.% CaCl_2_ mix is calculated to be approximately 0.7–0.8% by mass of cement, which is close to or slightly above the corrosion threshold (0.4–0.6% for reinforced concrete in many standards). Therefore, while the mechanical results are promising, it is strongly emphasized that this material is not recommended for reinforced concrete structures, especially those exposed to marine or humid environments. Instead, its potential application in unreinforced elements (e.g., pavements, blocks, or non-structural fillers) or in combined use with corrosion inhibitors is proposed.

## 4. Materials and Methods

### 4.1. Materials

The cement used in this experiment was P·I 42.5 Portland cement (PC), produced by Liaoning Fushun Cement Co., Ltd. (Fushun, China), and Fushun Aosai Technology Co., Ltd. (Fushun, China) The chemical composition of the cement is presented in Table 1. The sewage sludge adopted was collected from Nanning Huahong Mingyang Sewage Treatment Co., Ltd., Nanning, China. Sulfuric acid and CaCl_2_ (reagent grade) were supplied by Aladdin Industrial Corporation (Shanghai, China). Laboratory-grade distilled water was used in all experiments.

### 4.2. Sludge Pretreatment

**Figure 12:**
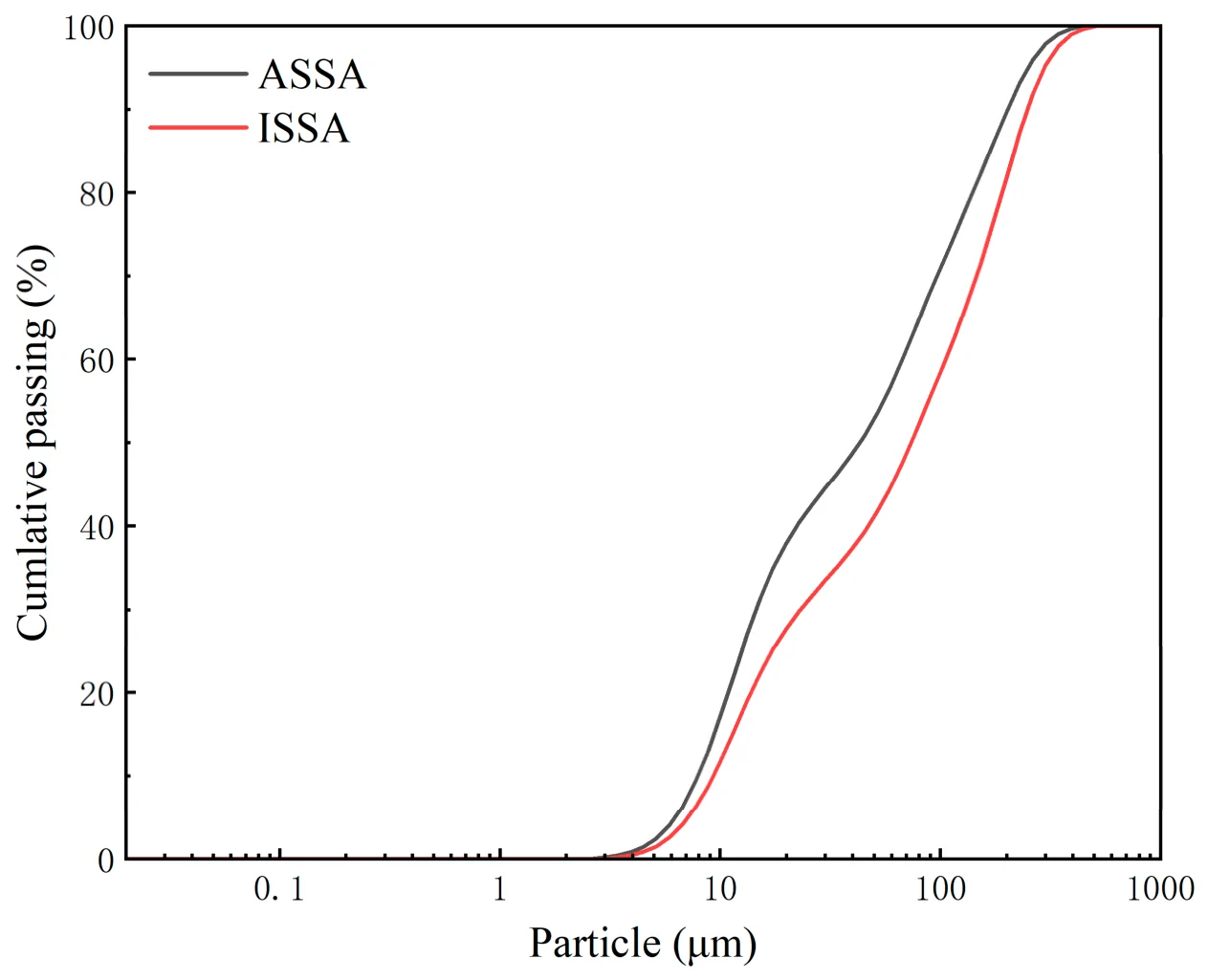
Particle size distribution of ASSA and ISSA.

**Figure 13:**
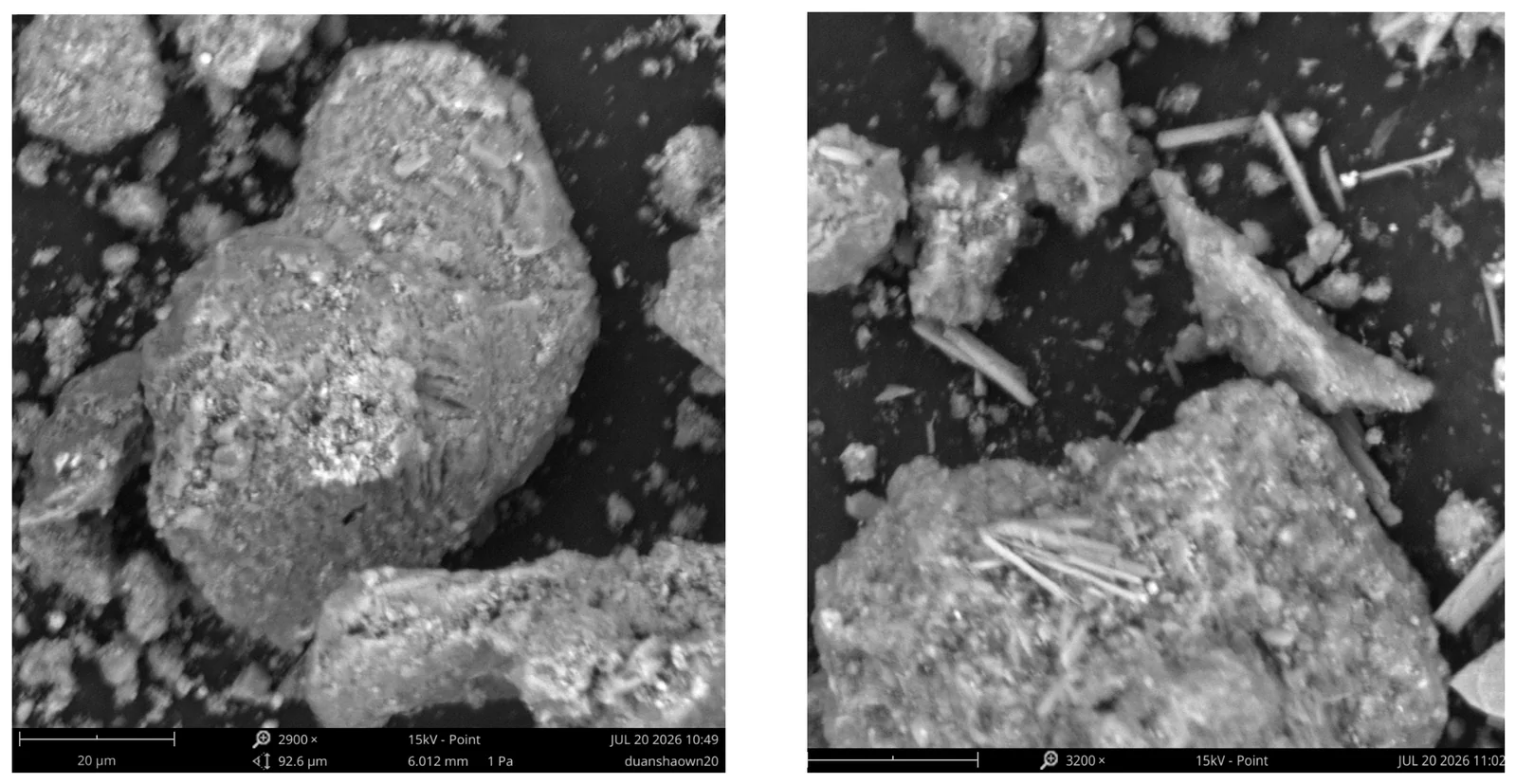
Morphologies of ASSA and ISSA.

According to the review conducted by C. J. Lynn et al. [15] on the research progress of sludge ash, the sludge ash obtained through calcination at 800 °C has the highest activity. Therefore, the raw sludge was dried at 110 °C for 24 h. Subsequently, it was coarsely ground using a crusher, and the ground sludge was calcined at a high temperature in a muffle furnace at 800 °C for 2 h. Furthermore, based on the optimal experimental conditions for extracting phosphorus using sulfuric acid as mentioned by L. F. Xu et al. [46], we also carried out an acid washing treatment on the sludge ash. The specific steps are as follows: The calcined sludge was acid-leached using a 0.3 mol/L sulfuric acid solution at a liquid-to-solid ratio of 10:1. The mixture was shaken for 2 h. Afterwards, the mixture was filtered through a 0.45 μm aqueous filter membrane, and the filter residue was dried at 110 °C for 6 h. It was then ground in a powder mill for 3 min, and finally the ASSA used in this experiment was obtained. The acid-leached sludge ash was tested to investigate the effect of acid leaching pretreatment on the chemical composition of the sludge ash (see Table 2). The specific surface areas of ISSA and ASSA, determined by the Blaine air permeability method, were 340.79 m^2^/kg and 327.68 m^2^/kg, respectively. And the particle size distributions of ISSA and ASSA measured by laser granulometry are shown in Figure 12. As can be seen from Figure 12, the mean particle size of ISSA is 73.75 μm, while that of ASSA is 43.08 μm. Figure 13 shows the morphologies of ISSA and ASSA, which reflect that ISSA exhibits an irregular shape and contains a large amount of open pores. Furthermore, ASSA also exhibits an irregular shape and the size is smaller than ISSA. In addition, the surface of ASSA has more open pores.

The acid leaching efficiency of sulfuric acid was determined by comparing the mass percentage of P_2_O_5_ before and after acid leaching. The P_2_O_5_ content in ASSA decreased from the original 22% to 6.5%, with a removal efficiency of 70%, which is consistent with the results reported in previous studies [39].

### 4.3. Sample Preparation

The experiment was divided into two types of samples: cement paste and mortar. The cement paste was used for testing the setting time and volume stability, heat of hydration and microstructure, while the mortar was used for evaluating the mechanical properties. The water–cement ratio is not fixed but is determined through multiple experiments to obtain the water requirement of normal consistency. The specific process includes: Before pouring the cement paste samples, the powder materials were mixed for 30 min using a laboratory-scale mixer to ensure complete blending. Then, the dry powders and the mixed liquid were stirred at 140 rpm for 120 s using a paste mixer, followed by a 15 s rest period, and during this period a scraper was used to transfer the paste on the mixing paddle into the mixer. And then further stirring at 285 rpm for 2 min followed. And finally it was poured into the test mold. The specific mix ratio of the cement paste system is shown in Table 3.

In addition, due to the numerous ISSA pores found through SEM observation, water can be adsorbed into these pores. Therefore, in order to ensure the good fluidity of the cement paste, a water-to-cement ratio of 0.5 was used to measure the heat of hydration and microstructure analysis. And this approach avoids experimental errors caused by variations in water content. At each testing age, the samples are removed from the water curing chamber, samples for X-ray diffraction (XRD), thermogracimetric analysis (TGA), fourier transform infrared spectrometer (FTIR) analysis, and scanning electron microscope (SEM) analysis. The hardened samples are crushed into fragments with a particle size of 5 mm, and then immersed in anhydrous ethanol for 24 h to terminate hydration, and finally vacuum-dried at 60 °C.

For the compressive strength tests, the mortar mixture followed the standard mix design specified in GB/T 17671-2021 [46], with a binder-to-sand-to-water ratio of 1:3:0.5. The ISSA-blended cement mix proportions are presented in Table 4. This standardized ratio was selected to ensure comparability with benchmark results in the literature and to maintain consistency with national testing protocols. The dry components were initially blended in a mortar mixer for 4 min at a low constant speed. Subsequently, the fine aggregate was incorporated and the blending continued for another 3 min. Following this, the premixed liquid solution was introduced into the mixture, and the entire batch was mixed for an additional 4 min. Upon completion of the mixing process, the fresh mortar was poured into 40 mm × 40 mm × 40 mm cubic molds and then conditioned for 24 h under controlled environmental conditions (20 ± 2 °C and 95% ± 2% relative humidity). After demolding, all specimens were transferred to a water bath for further curing until the designated testing ages were achieved.

### 4.4. Test Method

#### 4.4.1. Setting Time

According to the mix ratio in Table 3 and the sample preparation process, the samples that have already reached the normal consistency will undergo the tests for setting time and soundness. The first measurement was conducted 30 min after the cement was mixed with water. For each test, the mold was positioned under the initial setting needle, the needle was lowered to contact the paste surface, the screw was tightened for 1–2 s, and then suddenly released to allow the needle to penetrate vertically and freely into the paste. The scale reading was recorded when the needle stopped sinking or 30 s after its release. As the initial setting time approached, the test was repeated at intervals of 5 min or less. The cement paste was considered to have reached its initial set when the needle penetrated to a point 4 ± 1 mm from the bottom of the glass plate. At this point, the measurement was repeated immediately to confirm the result, and the initial setting time was recorded as the time elapsed from the addition of water to the cement until the initial set was achieved, expressed in minutes. Immediately after confirming the initial setting time, the mold containing the paste was carefully removed from the glass plate by a sliding motion, inverted 180° with the larger diameter end facing upward, and placed back on the glass plate in the moist curing cabinet. The final setting needle was then fitted to the apparatus. As the final setting time approached, the test was repeated at intervals of 15 min or less at different points on the specimen. The cement paste was considered to have reached its final set when the needle penetrated only 0.5 mm into the specimen, meaning that the annular attachment no longer left a visible impression on the surface. To confirm this endpoint, measurements were performed at two additional, different positions on the specimen, and the final setting time was recorded as the time elapsed from the addition of water to the cement until the final set was achieved, expressed in minutes. The setting time of the samples was determined in accordance with the Chinese National Standard Test Methods for Water Requirement of Normal Consistency, Setting Time and Soundness of the Cement.

#### 4.4.2. Compressive Strength

The compressive strength test is conducted using a 10 kN universal compression testing machine, with a load rating of 0.5 kN/s, on samples aged 3 d, 7 d, and 28 d. Three samples are tested, and their average value is taken as the compressive strength result of the specimens. This follows the Chinese National Standard Method of Test for Strength of Cement Mortars (GB/T 17671-2021) [46].

#### 4.4.3. Isothermal Calorimetry

Isothermal calorimetry tests were conducted using a TAM Air isothermal calorimeter (TA Instruments, New Castle, DE, USA) at a constant temperature of 20 °C to characterize the heat flow and cumulative hydration heat of the binder. Prior to measurement, all raw materials (binder powder and deionized water) were preconditioned at 20 °C for a minimum of 24 h to eliminate interference from initial temperature deviations. One day prior to formal testing, deionized water used as the reference sample was loaded into an ampoule and placed in the reference channel of the calorimeter. Formal tests were initiated only after the instrument’s temperature baseline had reached stable equilibrium.

Cement paste was prepared at a water-to-binder ratio (*w*/*c*) of 0.5, with a total paste mass of approximately 50 g per batch [13]. A high-speed electric mixer was employed to homogenize the paste at 500 rpm for 2 min. Immediately after mixing, approximately 8 g of homogeneous paste was transferred into a 20 mL ampoule using a Pasteur dropper. The ampoule was then sealed and rapidly inserted into the measuring channel, and data acquisition was launched with a total test duration of 72 h.

#### 4.4.4. XRD

The samples to be tested were ground into powder using a mortar and passed through a 200-mesh sieve. For cement hydration samples, hydration was first terminated by immersion in ethanol, followed by drying before grinding. The samples were scanned using an X-ray diffractometer over an angular range of 5–80° at a scanning speed of 10° per minute. After testing, phase analysis of the results was performed using Jade 6 software.

#### 4.4.5. FTIR

The samples were mixed with potassium bromide (KBr) at a mass ratio of 1:100 to 1:200 using the KBr pellet method, and the mixture was pressed into pellets. Functional group structural analysis of the sample powder was conducted using a GD26-FTIR-650 Fourier transform infrared spectrometer (PerkinElmer, Waltham, MA, USA) over a wavenumber range of 500–4000 cm^−1^.

#### 4.4.6. MIP

MIP measurements were carried out using an AutoPore IV 9510 high-performance automatic mercury porosimeter (Micromeritics, GA, USA), based on the principle that the volume of mercury intruding into porous media depends on the applied pressure. The maximum pressure was 33,000 psi, and the pore size measurement range was 50–1000 μm.

#### 4.4.7. TG-DSC

The grinding requirements for the test samples were consistent with those for XRD testing. A 5–10 mg sample was weighed and placed in an alumina crucible, then heated from 50 °C to 800 °C at a rate of 10 °C/min under a nitrogen atmosphere.

#### 4.4.8. SEM/EDS

The samples to be tested were ground into powder using a mortar and passed through a 200-mesh sieve. Scanning electron microscopy and energy-dispersive X-ray spectroscopy analyses were performed using a HELIOS NanoLab 600i microscope (FEI, Lake Oswego, OR, USA). Prior to testing, the samples were sputter-coated with gold under vacuum conditions.

## 5. Conclusions

This project studied the influence of different dosages of CaCl_2_ on the performance of ASSA–cement paste and obtained the following results:

(1) As the proportion of ASSA added increases, the setting time of ASSA–cement gradually prolongs, and the compressive strength correspondingly decreases. In contrast, in the group with added CaCl_2_, the setting time was significantly shortened as the CaCl_2_ dosage increased, and the extent of the shortening was positively correlated with the CaCl_2_ dosage. In addition, the incorporation of CaCl_2_ as a whole enhanced the compressive strength of cement, among which the effect of CaCl_2_ with a 2% dosage was the most significant, with a particularly obvious increase in compressive strength.

(2) The addition of CaCl_2_ promotes the hydration of C_3_S and C_2_S, increasing the formation of hydration products (AFt, CH, Kuzel’s salt and Friedel’s salt). At the same time, it shortens the induction period and increases the intensity of the main hydration peak. SEM analysis also shows that the addition of CaCl_2_ is conducive to the hydration of cement, making the structure more compact and reducing the unhydrated products, which is consistent with the changes in compressive strength and setting time.

(3) Compared with the control group without CaCl_2_ addition, the addition of 2% CaCl_2_ can refine the pore size, reduce the proportion of medium capillary pores, and simultaneously increase the volume proportion of fine and coarse mesoporous pores. Their content is significantly higher than that of ASSA–cement without CaCl_2_ addition. By refining the pore size, the compressive strength of the cement can be enhanced. When the content of CaCl_2_ was greater than 2%, a large number of voids and cracks were found in the microstructure, which was not conducive to the development of strength and thus led to a decrease in the compressive strength in the later stage.

(4) Beyond the mechanistic findings, this study offers several practical implications for the sustainable management of SSA in cement-based materials. First, the proposed acid washing pretreatment serves a dual function: it effectively removes the retarding phosphorus species from ISSA, while simultaneously generating a phosphorus-rich acidic leachate that can be harvested for agricultural or industrial phosphate recovery. This transforms a waste treatment challenge into a resource recovery opportunity, aligning with circular economy principles. Second, the identified optimal dosage of 2% CaCl_2_ provides a straightforward and cost-effective strategy to offset the strength loss associated with ASSA incorporation. Given that CaCl_2_ is a widely available industrial by-product, its application in this context does not significantly increase material costs, thereby facilitating potential field adoption.

(5) Despite the promising results, several limitations of this study should be acknowledged, which in turn outline worthwhile avenues for future investigation. The hydration mechanism and mechanical performance were primarily evaluated under laboratory-controlled conditions (curing at standard temperature and humidity). The long-term durability performance—particularly carbonation resistance, sulfate attack, and reinforcement corrosion risk in chloride-rich environments—has not yet been systematically assessed. A comprehensive durability assessment (including free chloride profiling, carbonation-induced chloride release, and corrosion current monitoring) is currently underway in ongoing research in this field and will be reported separately.

## Figures and Tables

**Table 1:** Chemical composition of clinker.

Oxide	Percentage/%	Anhydrous ph2.	Percentage/%
SiO_2_	22.11	/	/
Al_2_O_3_	4.43	C_3_S	54.58
Fe_2_O_3_	3.13	C_2_S	21.85
CaO	62.38	C_3_A	7.32
MgO	2.28	C_4_AF	12.24
SO_3_	2.62	Other	4.01
f-CaO	0.78	/	/

**Table 2:** Main chemical compositions of SSA and ASSA samples.

Samples	Na_2_O	MgO	Al_2_O_3_	SiO_2_	P_2_O_5_	SO_3_	CaO	TiO_2_	Fe_2_O_3_
ISSA	0.72	1.83	10.76	25.86	15.95	1.95	21.79	1.32	15.75
ASSA	0.59	1.27	16.75	34.46	3.53	4.26	7.65	1.52	26.96

**Table 3:** ISSA-blended cement paste mix ratio table.

ID	Cement/g	ISSA/g	ASSA/g	CaCl_2_/g	Water/g
T1C0	405	45	/	0	115
S1C0	405	/	45	0	115
S1C1	405	/	45	4.5	112
S1C2	405	/	45	9	111
S1C3	405	/	45	13.5	117
T2C0	360	90	/	0	115
S2C0	360	/	90	0	113
S2C1	360	/	90	4.5	112
S2C2	360	/	90	9	118
S2C3	360	/	90	13.5	112

Notes: T and S denote calcined and acid-washed sewage sludge ash, respectively. The digit following T/S indicates sludge ash dosage (1 = 10 wt%, 2 = 20 wt%); C stands for calcium chloride, with its trailing number representing mass dosage (0 = no addition, 1–3 = 1–3 wt%).

**Table 4:** ISSA-blended cement mortar mix ratio table.

ID	Cement/g	ISSA/g	ASSA/g	Sand/g	CaCl_2_/g	Water/g
T1C0	405	45	/	1350	0	225
S1C0	405	/	45	1350	0	225
S1C1	405	/	45	1350	4.5	225
S1C2	405	/	45	1350	9	225
S1C3	405	/	45	1350	13.5	225
T2C0	360	90	/	1350	0	225
S2C0	360	/	90	1350	0	225
S2C1	360	/	90	1350	4.5	225
S2C2	360	/	90	1350	9	225
S2C3	360	/	90	1350	13.5	225

Notes: T and S denote calcined and acid-washed sewage sludge ash, respectively. The digit following T/S indicates sludge ash dosage (1 = 10 wt%, 2 = 20 wt%); C stands for calcium chloride, with its trailing number representing mass dosage (0 = no addition, 1–3 = 1–3 wt%).

## Data Availability

Data is contained within the article.

## Abbreviations

The following abbreviations are used in this manuscript:

SSA	Sewage sludge ash
ASSA	Acid-washed sewage sludge ash
ISSA	Incinerated sewage sludge ash
PC	Portland cement
T1C0	10 wt% calcined sewage sludge ash without calcium chloride
S1C0	10 wt% acid-washed sewage sludge ash without calcium chloride
S1C1	10 wt% acid-washed sewage sludge ash with 1 wt% calcium chloride
S1C2	10 wt% acid-washed sewage sludge ash with 2 wt% calcium chloride
S1C3	10 wt% acid-washed sewage sludge ash with 3 wt% calcium chloride
T2C0	20 wt% aalcined sewage sludge ash without calcium chloride
S2C0	20 wt% acid-washed sewage sludge ash without calcium chloride
S2C1	20 wt% acid-washed sewage sludge ash with 1 wt% calcium chloride
S2C2	20 wt% acid-washed sewage sludge ash with 2 wt% calcium chloride
S2C3	20 wt% acid-washed sewage sludge ash with 3 wt% calcium chloride

## References

[1] Tuncer M.İ., Başyiğit C., Davraz M. (2025). The effect of sewage sludge ash as a mineral admixture on concrete strength. Constr. Build. Mater..

[2] Zhou J., Chen S., Das A.K. (2025). Application of sludge as a sustainable material in building materials: A comprehensive review. J. Build. Eng..

[3] Sidhu A.S., Siddique R., Singh G. (2024). Review on the effect of sewage sludge ash on the properties of concrete. Constr. Build. Mater..

[4] Terán-Cuadrado G., Tahir F., Nurdiawati A., Almarshoud M.A., Al-Ghamdi S.G. (2024). Current and potential materials for the low-carbon cement production: Life cycle assessment perspective. J. Build. Eng..

[5] Guo Y., Luo L., Liu T., Hao L., Li Y., Liu P., Zhu T. (2024). A review of low-carbon technologies and projects for the global cement industry. J. Environ. Sci..

[6] Costa R.P., De Medeiros M.H.G., Martinez E.D.R., Quarcioni V.A., Suzuki S., Kirchheim A.P. (2022). Effect of soluble phosphate, fluoride, and pH in Brazilian phosphogypsum used as setting retarder on Portland cement hydration. Case Stud. Constr. Mater..

[7] Basri S., Oruganti R.K., Panda T.K., Bhattacharyya D. (2025). Beyond conventional approaches: Sustainable valorization of sewage sludge—Challenges and opportunities. Sustain. Mater. Technol..

[8] Bhusal M.R., Fonseka C.W., Muthukumaran S., Joseph P., Sandanayake M. (2025). Water treatment sludge as a partial cement replacement material: Preliminary investigations and future perspectives. Innov. Infrastruct. Solut..

[9] Mejdi M., Saillio M., Chaussadent T., Divet L., Tagnit-Hamou A. (2020). Hydration mechanisms of sewage sludge ashes used as cement replacement. Cem. Concr. Res..

[10] Mahutjane T.C., Tchadjié L.N., Sithole T.N. (2023). The feasibility of utilizing sewage sludge as a source of aluminosilicate to synthesise geopolymer cement. J. Mater. Res. Technol..

[11] Xia Y., Liu Y., Wang L., Song Z., Sun C., Zhao Y., Lu S., Yan J. (2023). Value-added recycling of sludge and sludge ash into low-carbon construction materials: Current status and perspectives. Low-Carbon Mater. Green Constr..

[12] Chen Z., Poon C.S. (2017). Comparative studies on the effects of sewage sludge ash and fly ash on cement hydration and properties of cement mortars. Constr. Build. Mater..

[13] Xia Y., Liu M., Zhao Y., Chi X., Guo J., Du D., Du J. (2023). Hydration of ternary blended cements with sewage sludge ash and limestone: Hydration mechanism and phase assemblage. Constr. Build. Mater..

[14] Sugiura Y., Ishikawa K., Onuma K., Makita Y. (2019). PO4 adsorption on the calcite surface modulates calcite formation and crystal size. Am. Mineral..

[15] Chen M., Blanc D., Gautier M., Mehu J., Gourdon R. (2013). Environmental and technical assessments of the potential utilization of sewage sludge ashes (SSAs) as secondary raw materials in construction. Waste Manag..

[16] Lynn C.J., Dhir R.K., Ghataora G.S., West R.P. (2015). Sewage sludge ash characteristics and potential for use in concrete. Constr. Build. Mater..

[17] Danish A., Ozbakkaloglu T. (2022). Greener cementitious composites incorporating sewage sludge ash as cement replacement: A review of progress, potentials, and future prospects. J. Clean. Prod..

[18] Boughanmi S., Labidi I., Megriche A., Tiss H., Nonat A. (2018). Does phosphorus affect the industrial Portland cement reactivity?. Constr. Build. Mater..

[19] Holanda F.D.C., Schmidt H., Quarcioni V.A. (2017). Influence of phosphorus from phosphogypsum on the initial hydration of portland cement in the presence of superplasticizers. Cem. Concr. Compos..

[20] Huang Y., Qian J., Liu C., Liu N., Shen Y., Ma Y., Sun H., Fan Y. (2017). Influence of phosphorus impurities on the performances of calcium sulfoaluminate cement. Constr. Build. Mater..

[21] Świerczek L., Cieślik B.M., Konieczka P. (2018). The potential of raw sewage sludge in construction industry—A review. J. Clean. Prod..

[22] Chang Z., Long G., Xie Y., Zhou J.L. (2022). Pozzolanic reactivity of aluminum-rich sewage sludge ash: Influence of calcination process and effect of calcination products on cement hydration. Constr. Build. Mater..

[23] Guo X., Yuan S., Xu Y., Qian G. (2022). Effects of phosphorus and iron on the composition and property of Portland cement clinker utilized incinerated sewage sludge ash. Constr. Build. Mater..

[24] Raniro H.R., Serrano-Gomez J., Mort H.L., Kalpakchiev T., Kooij J., Zhao Y., Valença R.M., Magaya S., Guerrero-Esquivel A.J., Korving L. (2025). Overcoming recycling barriers to transform global phosphorus management. Nat. Rev. Earth Environ..

[25] Fang Y., Liu A., Li N., Jiang N., Qian G., Xu Y. (2025). Phosphorus recovery from Fe/Al-rich sewage sludge ash: Combining wet and thermochemical methods. Sep. Purif. Technol..

[26] Zhang X., Sun S., Han X., Liu H., Shu S. (2025). Phosphorus recovery from urban wastewater treatment in China: Current status, future potential and a roadmap for sustainable development. J. Water Process Eng..

[27] Kasina M. (2022). The assessment of phosphorus recovery potential in sewage sludge incineration ashes—A case study. Environ. Sci. Pollut. Res..

[28] Boniardi G., Turolla A., Fiameni L., Gelmi E., Malpei F., Bontempi E., Canziani R. (2021). Assessment of a simple and replicable procedure for selective phosphorus recovery from sewage sludge ashes by wet chemical extraction and precipitation. Chemosphere.

[29] Ottosen L.M., Thornberg D., Cohen Y., Stiernström S. (2022). Utilization of acid-washed sewage sludge ash as sand or cement replacement in concrete. Resour. Conserv. Recycl..

[30] Liang S., Yang L., Chen H., Yu W., Tao S., Yuan S., Xiao K., Hu J., Hou H., Liu B. (2021). Phosphorus recovery from incinerated sewage sludge ash (ISSA) and reutilization of residues for sludge pretreated by different conditioners. Resour. Conserv. Recycl..

[31] Kappel A., Viader R.P., Kowalski K.P., Kirkelund G.M., Ottosen L.M. (2018). Utilisation of electrodialytically treated sewage sludge ash in mortar. Waste Biomass Valorization.

[32] Cong X., Lu S., Gao Y., Yao Y., Elchalakani M., Shi X. (2020). Effects of microwave, thermomechanical and chemical treatments of sewage sludge ash on its early-age behavior as supplementary cementitious material. J. Clean. Prod..

[33] Xia Y., Liu M., Zhao Y., Ma X. (2022). Microstructure of Portland cement blended with high dosage of sewage sludge ash activated by Na_2_SO_4_. J. Clean. Prod..

[34] Fu J., Bligh M.W., Shikhov I., Jones A.M., Holt C., Keyte L.M., Moghaddam F., Arns C.H., Foster S.J., Waite T.D. (2021). A microstructural investigation of a Na_2_SO_4_ activated cement-slag blend. Cem. Concr. Res..

[35] Xu Y., Chen J., Yang F., Fang Y., Qian G. (2021). Transformation of phosphorus by MgCl_2_ and CaCl_2_ during sewage sludge incineration. Environ. Sci. Pollut. Res..

[36] Zhou Y., Lu J., Li J., Cheeseman C., Poon C.S. (2021). Effect of NaCl and MgCl_2_ on the hydration of lime-pozzolan blend by recycling sewage sludge ash. J. Clean. Prod..

[37] Zhao Q., Lv T., Liang H., Zhang J., Zhang J. (2023). Enhancement of sintered sludge ash-modified cement paste with CaSO_4_ and CaCl_2_. Constr. Build. Mater..

[38] Li M.G., Tan H.T., Zhang J.J., Deng X.F., Kong X.H., Chen P., Jian S.W., He X.Y., Yang J. (2023). Enhancement in compressive strength of carbide slag activated ground granulated blast furnace slag by introducing CaCl_2_ and NaCl. Constr. Build. Mater..

[39] Shi C.J., Qian J.S. (2001). Activated blended cement containing high volume coal fly ash. Adv. Cem. Res..

[40] Fang L., Li J., Guo M.Z., Cheeseman C.R., Tsang D.C.W., Donatello S., Poon C.S. (2018). Phosphorus recovery and leaching of trace elements from incinerated sewage sludge ash (ISSA). Chemosphere.

[41] Yum W.S., Jeong Y., Yoon S., Jeon D., Jun Y., Oh J.E. (2017). Effects of CaCl_2_ on hydration and properties of lime(CaO)-activated slag/fly ash binder. Cem. Concr. Compos..

[42] Xia Y., Liu M., Zhao Y., Chi X., Guo J., Du D., Du J. (2023). Hydration mechanism and phase assemblage of blended cement with iron-rich sewage sludge ash. J. Build. Eng..

[43] Zhang Z., Yang F., Liu J.-C., Wang S. (2020). Eco-friendly high strength, high ductility engineered cementitious composites (ECC) with substitution of fly ash by rice husk ash. Cem. Concr. Res..

[44] Juenger M.C.G., Monteiro P.J.M., Gartner E.M., Denbeaux G.P. (2005). Soft X-ray microscope investigation into the effects of calcium chloride on tricalcium silicate hydration. Cem. Concr. Res..

[45] Xu L.F., Chen Y., Liu M., Fu X. (2020). Comparison of Phosphorus Extraction from Sludge Incinerated Bottom Ash and Sludge Incinerated Fly Ash Using Sulfuric Acid. Environ. Eng. Sci..

[46] (2021). Test Method of Cement Mortar Strength (ISO Method) (National Standard of China).

